# 3D vasculature segmentation using localized hybrid level-set method

**DOI:** 10.1186/1475-925X-13-169

**Published:** 2014-12-16

**Authors:** Qingqi Hong, Qingde Li, Beizhan Wang, Yan Li, Junfeng Yao, Kunhong Liu, Qingqiang Wu

**Affiliations:** Software School, Xiamen University, 361005 Xiamen, China; Department of Computer Science, University of Hull, HU6 7RX Hull, UK; Management School, Xiamen University, 361005 Xiamen, China

**Keywords:** Segmentation, Vessel image, Level-set, Intensity inhomogeneity

## Abstract

**Background:**

Intensity inhomogeneity occurs in many medical images, especially in vessel images. Overcoming the difficulty due to image inhomogeneity is crucial for the segmentation of vessel image.

**Methods:**

This paper proposes a localized hybrid level-set method for the segmentation of 3D vessel image. The proposed method integrates both local region information and boundary information for vessel segmentation, which is essential for the accurate extraction of tiny vessel structures. The local intensity information is firstly embedded into a region-based contour model, and then incorporated into the level-set formulation of the geodesic active contour model. Compared with the preset global threshold based method, the use of automatically calculated local thresholds enables the extraction of the local image information, which is essential for the segmentation of vessel images.

**Results:**

Experiments carried out on the segmentation of 3D vessel images demonstrate the strengths of using locally specified dynamic thresholds in our level-set method. Furthermore, both qualitative comparison and quantitative validations have been performed to evaluate the effectiveness of our proposed model.

**Conclusions:**

Experimental results and validations demonstrate that our proposed model can achieve more promising segmentation results than the original hybrid method does.

## Background

The reconstruction of blood vessel plays an important role for many clinical tasks such as diagnosis of vessel diseases, surgery planning and blood flow simulation [1]. The first task to vessel reconstruction is to identify the vessel region from initial vessel images. This is usually achieved by using the technique of segmentation, which plays an important role in medical image processing. Although many segmentation techniques have been proposed for various image segmentation problems, blood vessel image segmentation still remains a challenging task [2], due to the high complexity of vessel branching and thinning geometry as well as the decrease in image contrast from the root of the vessel to its thin branches [1].

Among all the segmentation techniques, the deformable model (i.e. active contour) [3] is one of the most popular approaches during the past two decades. The basic idea of deformable model method is to evolve the initial curve/surface to the boundaries of target objects driven by the combination of internal forces determined by the geometry of the evolving curve and the external forces induced from the image [4]. Depending on the way the underlying curved contour is represented, the deformable active contour can be implemented either parametrically or implicitly [5]. In parametric models, active contours are explicitly represented in parametric forms in a Lagrangian framework [6]. A multitude of powerful methods based on parametrical deformable model have been proposed for the segmentation of medical image [7]. Klein et al. [8] presented a method for the extraction of vessel boundaries using deformable surface models represented by B-spline functions. However, the parameterized deformable models are difficult to handle the complex topological changes for the whole vasculatures during evolution, as reparameterization is a tough problem to be solved for parametric model [9]. On the other hand, active contours can be implicitly represented as level-set [10] function, which is embedded in higher dimensional spaces and evolves according to the Partial Differential Equation (PDE) in an Eulerian framework [6]. Compared with parametric model, level-set model is easier to perform shape operations, and adapt freely into complex topologies of objects. Therefore, various level-set based active models have been developed and applied to image segmentation. Some of the well-known models are the geodesic active contour model [11] that utilizes image gradient to stop evolving contours on the object boundaries, and Chan-Vese model [12] that solves the leakage problem by using region information of the target boundary for segmentation. There are also several localized active models [13, 14] introducing the local image fitting energy to extract the local image information been presented to improve the performance of the active contours. Furthermore, some techniques combining edge and region information [4, 15, 16] have been developed and applied to segmentation of medical imaging.

In the area of 3D vessel image segmentation, Lorigo et al. [17] proposed the ’CURVES’ algorithm to extract vessels by using a geodesic active contour based on the codimension two level set method [18]. Nain et al. [1] proposed a region-based geometric deformable model for MRA segmentation, which is driven by the combination of image statistics and shape information. Yan and Kassim [19] presented a segmentation scheme for the extraction of vasculature from MRA images by using capillary active contour. In [20], the vessel contours are segmented from the enhanced dataset by means of a level set evolution, which relies on the image intensity statistics to either expand or shrink the evolving contour.In [21], a shape functional is used to regularize the ‘CURVES’ and flux maximizing flow functional [22], which is claimed to be a promising tool to improve the efficiency of both techniques. Law and Chung [23] presented a vessel extraction approach based on a weighted local variance based edge detection scheme, which is effective for the segmentation of vessel image with low contrast edges. Recently, Wang and Jiang presented a nonparametric shape constrained algorithm for segmentation of coronary arteries in computed tomography images within the framework of active contours [24]. However, as most of these active contour models only take the edge information or region information of the image into account, they may have difficulty in correctly extracting tiny vessels from images, due to the low image contrast of the thin vessel branches.

In this paper, a localized hybrid level-set method for the segmentation of 3D vessel image is proposed. Our technique is inspired by Zhang et al.’s hybrid level-set model [4]. The hybrid model integrates both region and boundary information for the segmentation of medical images, in which the boundary information can help to detect the precise location of the target object and the region information can help to prevent the boundary leakage problem. However, the original hybrid model utilized a preset global threshold indicating the lower bound of target object to specify the term concerning regional information, which is not quite appropriate, especially for medical images with intensity inhomogeneity. In fact, intensity inhomogeneity occurs in many medical images [13], especially in vessel images, as the vessel branching is highly complex and the image contrast decreases from the root of the vessel to its thin branches [1]. On the other hand, our proposed localized hybrid level-set model utilizes the automatically calculated dynamic thresholds to indicate the lower bound of target object to specify the term concerning regional information instead of using a preset global threshold, which is crucial for the segmentation of vessel images [15]. In our proposed technique, the local intensity information is firstly embedded into a region-based contour model, and then incorporated into the level-set formulation of the geodesic active contour model. Compared with the global threshold based method, the use of locally specified dynamic thresholds enables the extraction of the local image information, which is essential for the segmentation of vessel images. Experimental results on 3D medical datasets and validations are presented to demonstrate the strengths of our localized hybrid level-setmethod.

## Methods

### Theory

In the proposed localized hybrid model, the local intensity information is firstly embedded into a region-based contour model, and then incorporated into the level-set formulation of the geodesic active contour model. Compared with original hybrid level-set model [4], our proposed model calculates the locally specified dynamic thresholds to indicate the lower bound of target object, instead of using a preset global threshold to specify the term concerning regional information.Consider a given image *I*:*Ω*→*R*, in which *Ω*⊂*R*^*n*^ (*n*=2 or 3) is the image domain, and let *ϕ*:*Ω*→*R* be a contour in the image domain. The proposed energy functional to be minimised is defined as:
1

where **u**∈*Ω*, and *α* and *β* are the preset weights to balance the two terms. *g*:[0,*∞*]→*R*^+^ is the decreasing function, such as *g*(*h*)=1*/*(1+*ch*^2^) with *c* controlling the scope, and *H*(*ϕ*) is the Heaviside function defined as:
2

*μ*(**u**)is the automatically calculated localized thresholds indicating the lower bound of target object. The definition of *μ*(**u**)is as below:
3

where *k*∈[0.5,1] is an adjustment coefficient, and *K*_*σ*_ is the Gaussian kernel function with a localization property, such as  with a scale parameter *σ*. The coefficient *k* is effective for preventing the active contours stopping evolving inside the target areas before reaching the boundary, which makes our proposed model more robust for the segmentation of 3D medical dataset. The first term of functional (1) defines the region term in our localized hybrid level-set model. The second term is equal to the geodesic active contour functional, which is the same as the second term of the original hybrid model. In addition to keeping the advantages of original hybrid model, the localized hybrid model is able to extract more accurate local image information by utilizing the locally specified dynamic thresholds *μ*(**u**) to indicate the lower bound of target object, which is essential for segmenting images with intensity inhomogeneity.

The associated PDE can be derived from the gradient descent flow [13] applied to functional (4)
4

### Implementation

The original Heaviside function is discontinuous and is unable to achieve smooth transition at the boundary. In practice, this problem is generally solved using a kind of smooth Heaviside function, which is usually called smooth step function or smooth unit step function. Depending on the problem of applications, different smooth step functions have been introduced to approximate smoothly the Heaviside function [25]. More recently, a piecewise polynomial smooth unit step functions is developed and used for geometric shape design, which can be considered as a generalization of the Heaviside unit step function [26].

In our implementation, we adopt the smooth function *H*_*ε*_(*s*) introduced in [12]
5

and the corresponding Dirac function *δ*_*ε*_(*s*) is defined as
6

Then, *H* in Equation (1) is replaced with *H*_*ε*_, and *δ* in Equation (4) is replaced with *δ*_*ε*_. In addition, in order to avoid problems of developing shocks, sharp or flat shapes during evolution, it is essential to reinitialize *ϕ* to be a signed distance function (SDF) [27]. And if *ϕ* is a SDF, then |∇*ϕ*|=1. Therefore, Equation (4) can be written as
7

In the proposed model, the temporal partial derivative *∂ϕ/∂t* is approximated by the forward difference scheme. Then, the approximation of Equation (4) can be expressed as [6]
8

where *ϕ*^(*k*+1)^ and *ϕ*^(*k*)^ denote the embedding function *ϕ* in the (*k*+1)^*th*^ and (*k*)^*th*^ iterations respectively, and *Δt* is the predefined time step. The difference equation (8) can also be rewritten as the following iteration
9

Finally, the principal steps in the original hybrid model [4] is adopted to update from *ϕ*^(*k*)^ to *ϕ*^(*k*+1)^:

1. Reinitialize *ϕ*^(*k*)^ to be SDF;
2. Calculate the localized thresholds *μ*(**u**) automatically according to Equation 3;
3. Compute the local region energy functional *δ*_*ε*_(*ϕ*^(*k*)^)(*I*−*μ*(**u**));
4. Update *ϕ*^(*k*)^ to *ϕ*^(*k*)^^′^ using *ϕ*^(*k*)^^′^=*ϕ*^(*k*)^+*Δtαδ*_*ε*_(*ϕ*^(*k*)^)(*I*−*μ*(**u**));
5. Reinitialize *ϕ*^(*k*)^^′^ to be SDF;
6. Update *ϕ*^(*k*)^^′^ to obtain *ϕ*^(*k*+1)^ via *ϕ*^(*k*+1)^=*ϕ*^(*k*)^^′^+*Δtβδ*_*ε*_(*ϕ*^(*k*)^)*div*(*g*(|∇*I*|)∇*ϕ*^(*k*)^).

## Results and discussion

### Results of our localized hybrid model

The proposed localized hybrid model has been applied to more than ten 3D medical datasets for the segmentation of vasculatures. The datasets are supplied by Intelligent Bioinformatics Systems Division, Institute of Automation, the Chinese Academy of Sciences, in the format of DICOM (Digital Imaging and Communications in Medicine). In all these experiments, the same parameters are used, namely, *Δt*=4.0, *α*=0.01, *β*=0.5, *σ*=3.0, *ε*=1.0, and *k*=0.9. In the following, we present some typical segmentation results as well as the Maximum Intensity Projections (MIPs) of the corresponding original datasets. MIPs are very useful to evaluate current 3D angiography images since the overall shapes and paths of the vessel can be clearly visualized [28].

**Figure 1:**
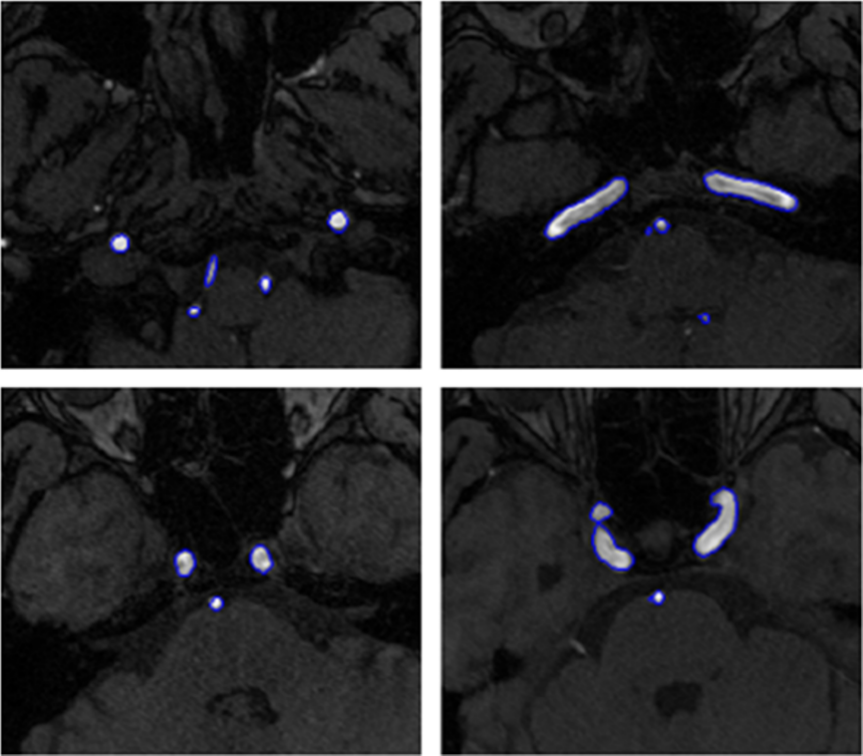
**Some 2D slices of the segmentation result on cerebral MRA dataset using the proposed localized hybrid technique.**

**Figure 2:**
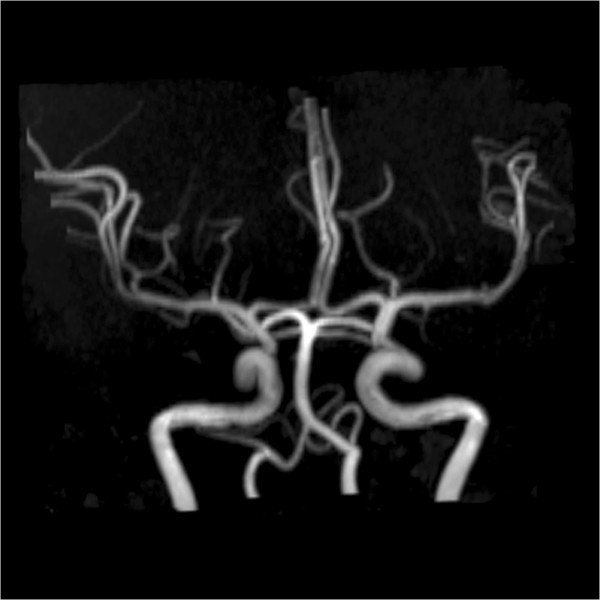
**Maximum Intensity Projection (MIP) of the cerebral MRA dataset.**

**Figure 3:**
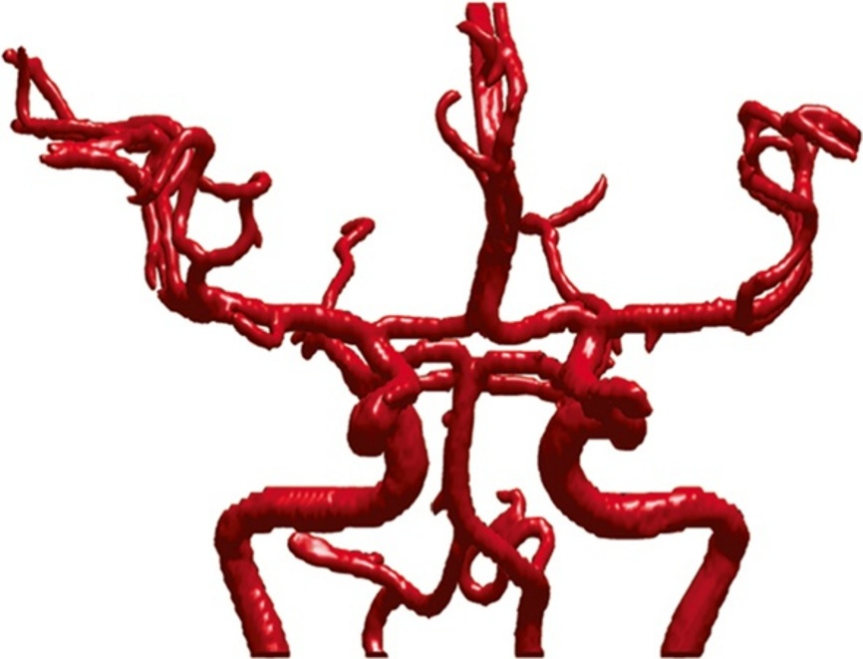
**3D vascular tree of the Segmentation results on cerebral MRA dataset using the proposed localized hybrid technique.**

The first example is the segmentation of cerebral vasculatures for 3D MRA images with a resolution of 352×448×114 and spacing of 0.49 *mm*×0.49 *mm*×0.80 *mm*. Figure 1 shows the segmentation results for some 2D slices of the dataset. As mentioned before, Magnetic Resonance (MR) images are typically intensity inhomogeneous. As can be seen from Figure 1, the intensity of the middle region is quite lower than that of other regions of the vessel. Owning to the utilization of locally specified dynamic thresholds, the proposed method can successfully depict the vessels contours (indicated by blue curves) in these images. Figure 2 shows the MIP of this dataset, and Figure 3 presents the segmentation result of whole complex cerebral vasculature successfully extracted from the 3D MRA dataset.

**Figure 4:**
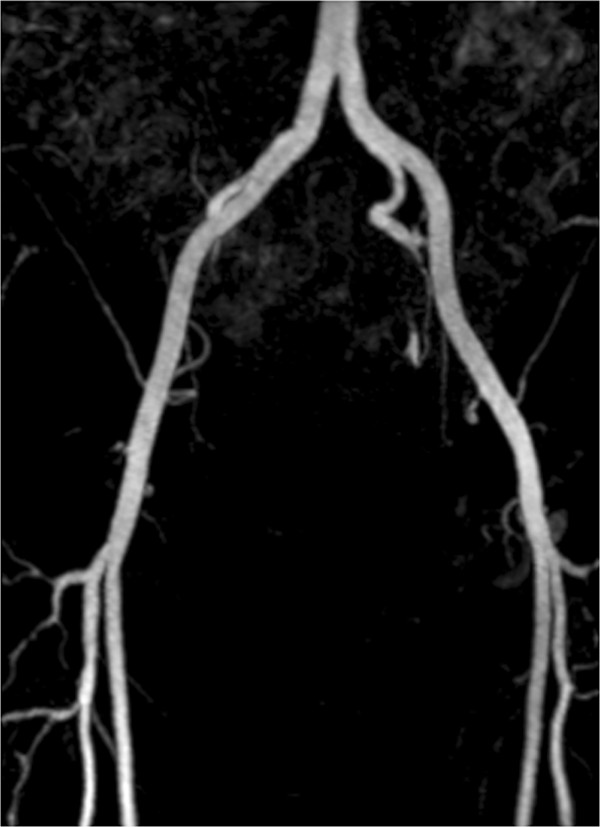
**Maximum Intensity Projection (MIP) of the MRA dataset for common iliac artery.**

**Figure 5:**
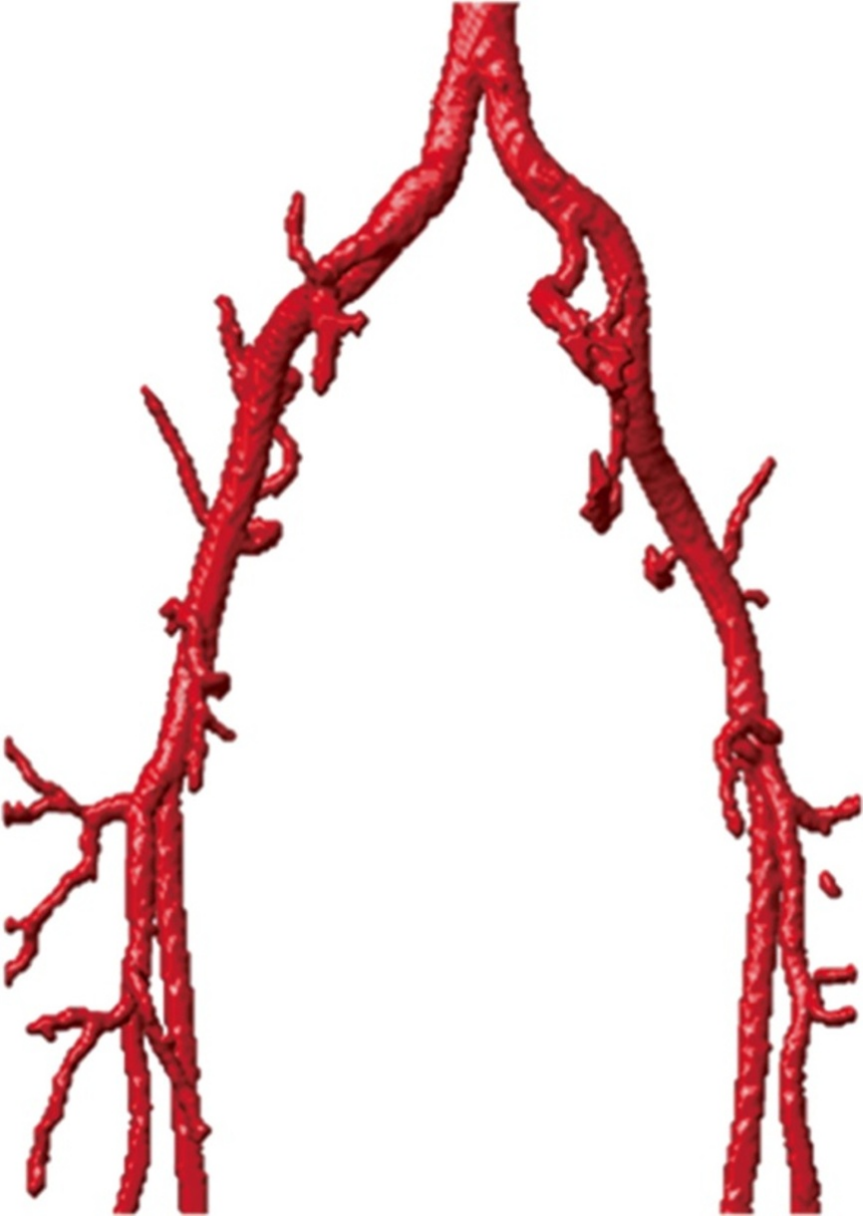
**3D vascular tree of the segmentation result on iliac artery MRA dataset using the proposed localized hybrid technique.**

The second example is the segmentation of the common iliac artery for 3D MRA images with a resolution of 384×384×72 and spacing of 1.0 *mm*×1.0 *mm*×1.5 *mm*. The MIP of this dataset is shown in Figure 4, while the segmentation result using our localized hybrid technique is shown in Figure 5. Although the raw dataset has great intensity inhomogeneity, the whole iliac artery is still successfully extracted.

**Figure 6:**
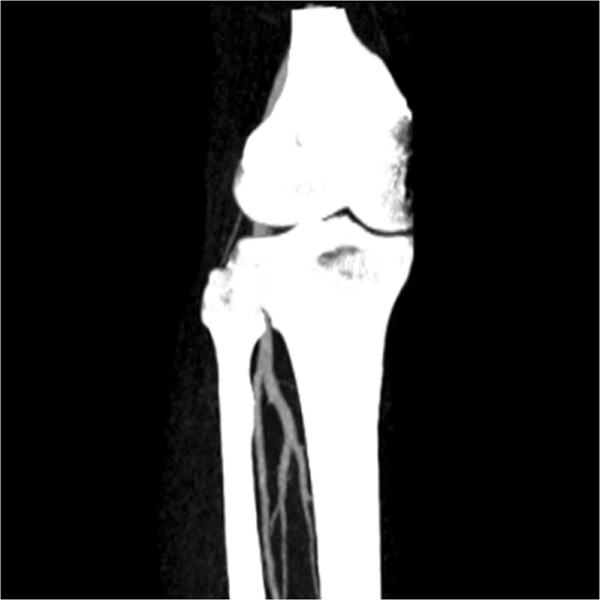
**Maximum Intensity Projection (MIP) of the CTA dataset for peripheral artery.**

**Figure 7:**
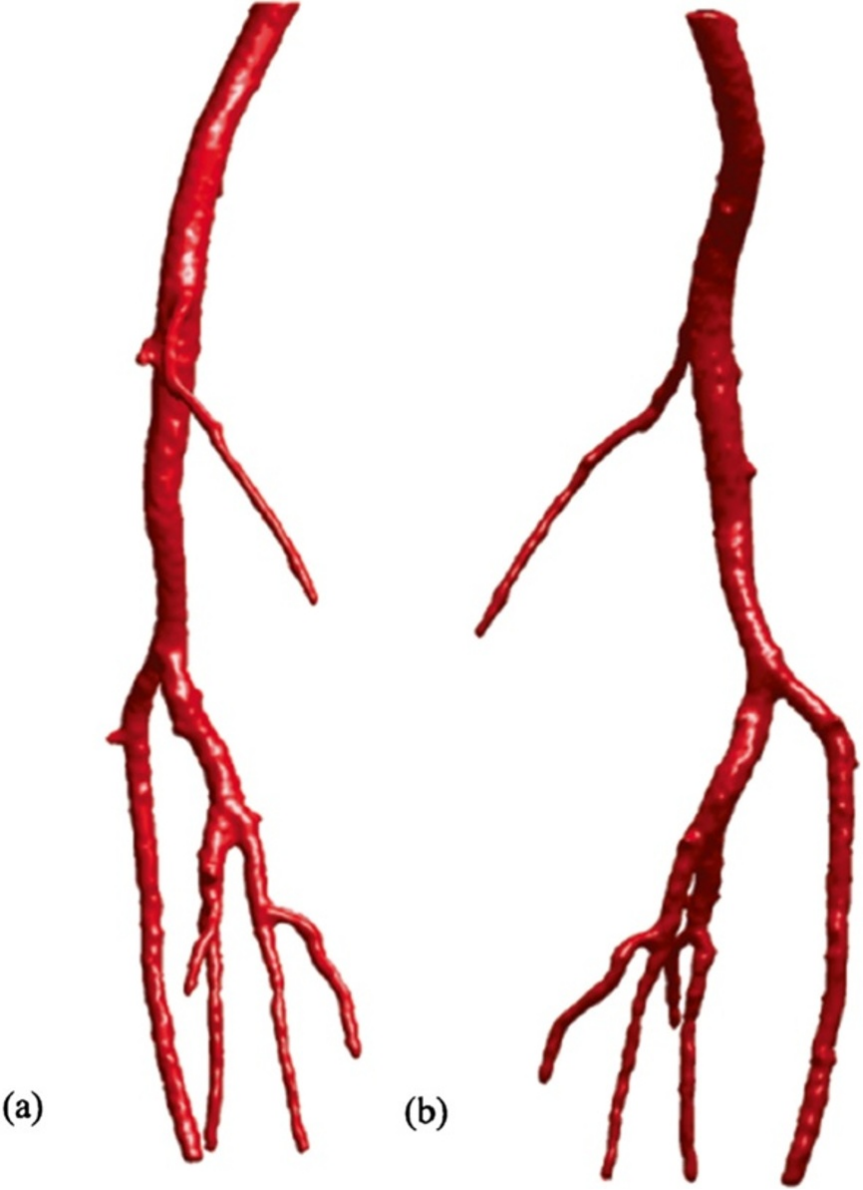
**3D vascular tree of the segmentation result on peripheral artery CTA dataset using the proposed localized hybrid technique.** **(a, b)** are the visualization of segmentation results rendered from different viewing angles.

Another example is the segmentation of peripheral artery for the 3D CTA images with a resolution of 512×512×240 and spacing of 0.83 *mm*×0.83 *mm*×1.0 *mm*. As can been seen from the MIP of this dataset (Figure 6), the visualization of vessel structures is greatly distracted by bone areas. However, our proposed model can still successfully extract the whole peripheral artery structures from the raw images despite of the distraction of bone structures (see Figure 7).

### Comparison with the original hybrid level-set model

In this section, we investigate why the original hybrid model may fail in the segmentation of real medical data with intensity inhomogeneity whereas our localized model works. According to our prior knowledge, the intensity (i.e. gray level) of most vessels in the first dataset (i.e. cerebral MRA images) is larger than 200. However, the intensity of some small vessels and their branches can be varied, for some of them can even be lower than 100. Therefore, for the original model, it is quite difficult to predefine the global threshold *μ* indicating the lower bound of gray level of target vessel. If the global threshold *μ* were set to be 200, it would be unable to extract the small vasculatures with intensity smaller than 200; if *μ* were set to be 100, the segmentation result could include some irrelevant objects, which would lead to inaccurate extraction of vasculatures.

**Figure 8:**
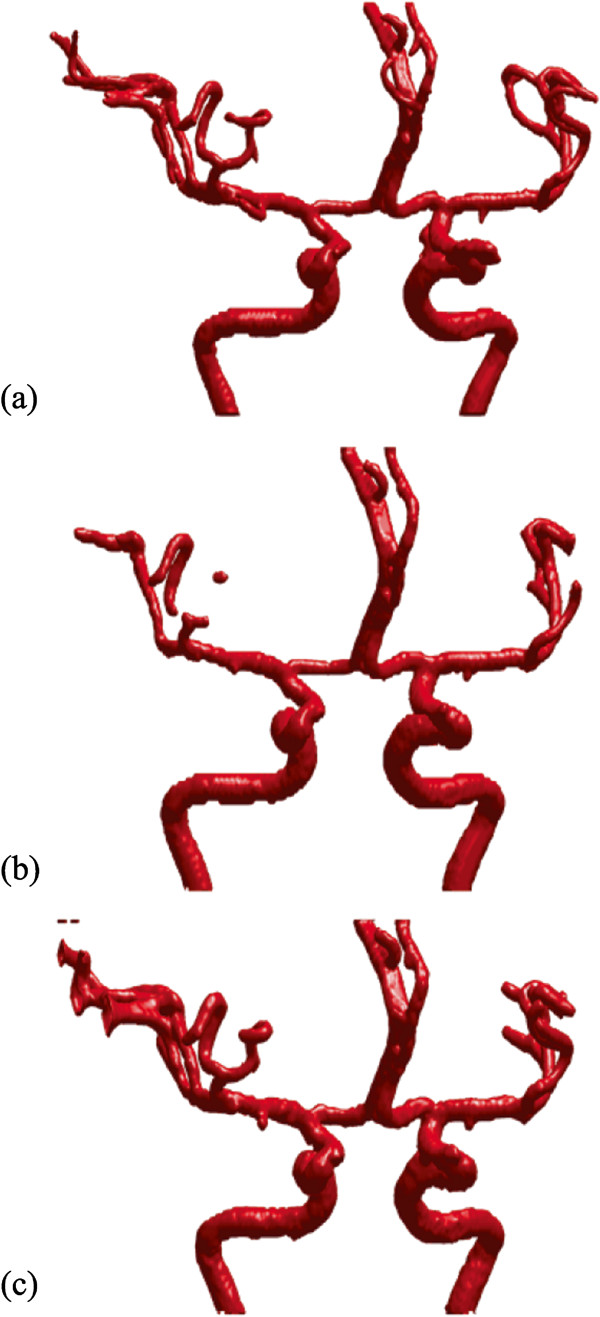
**Comparison of our localized model with the original model.** **(a)** Part of segmentation results using our localized model. **(b)** Part of segmentation results using the original model with *μ*=100. **(c)** Part of segmentation results using the original model with *μ*=200.

For the purpose of comparison, we use the same set of parameter values (please refer to the beginning of this section) for the common parameters of the original model and our localized model. In addition, we also set the same seed contours for both models. As can be seen from Figure 8(a), it is very clear that our method has the ability to extract small branches with satisfactory results. Figure 8(b) shows the segmented result of the original model with *μ*=200. In this case, it is unable to extract small branches with an intensity less than 200, which just provides us with the unconnected vessel tree. On the other hand, when *μ* is set as 100, the original hybrid model can produce a more connected vessel tree, but some of the individual branches become much thicker than expected, which can be seen clearly in Figure 8(c). In other words, when the value of *μ* is set too low in the original hybrid model, it may also lead to inaccurate segmentation of vasculatures.

**Figure 9:**
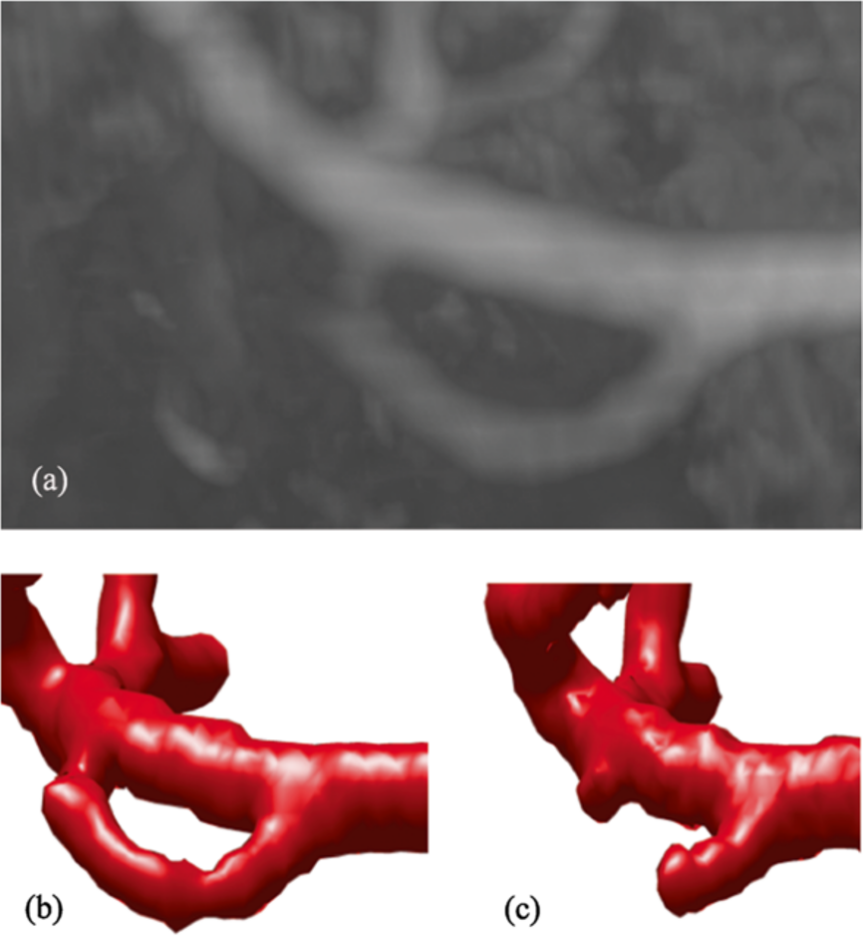
**Detailed comparison of the segmentation of thin vessels in low contrast situations. (a)** A detailed look at the MIP of the original 3D dataset. **(b)** Our localized hybrid model. **(c)** Original model with *μ*=100.

Moreover, the original hybrid model may be still unable to extract thin vessels in low contrast situations even though *μ* is set to be very low. As can be seen from Figure 9,(a) is a detailed look at the MIP of the 3D dataset, and the rectangular region in (b) indicates the successful extraction of thin vessels with low contrast using our localized model. Figure 9(c) shows the segmentation result using the original hybrid model with *μ*=100, which still cannot extract thin vessels.

In a word, this qualitative comparison indicates that our localized hybrid model can achieve a more satisfactory segmentation result of the vascular structures than the original model with different *μ*s.

### Validations

**Figure 10:**
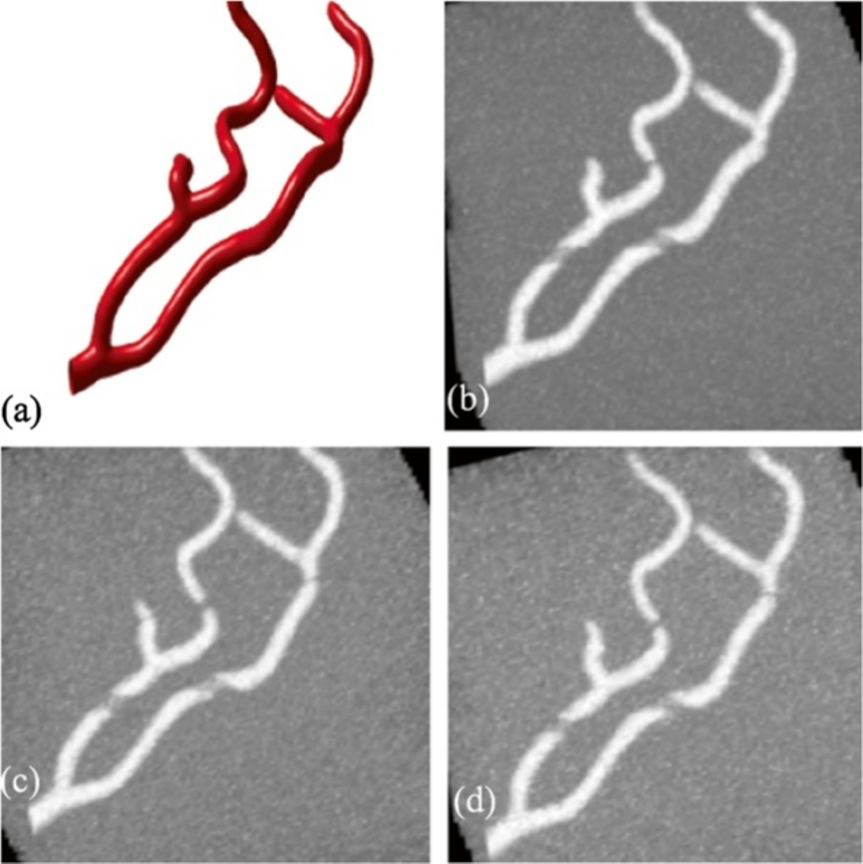
**The test dataset for validations. (a)** The known vessel pattern reconstructed from the real MRA images; **(b)-(d)** MIPs of corrupted data sets by Gaussian noise distributions with variances of 0.001, 0.01 and 0.015 respectively.

In this study, a test dataset as shown in Figure 10 with the resolution of 100×70×106 voxels is used to evaluate the performance of the proposed localized hybrid model. In this dataset, an actual vessel structure reconstructed from the real MRA images is chosen as the known vessel pattern for validation, which is shown in Figure 10(a). The maximum intensity value in the original dataset is 500, and the intensity value of vessel boundary is 250. Then the original dataset is corrupted by several zero-mean Gaussian noise levels. Furthermore, the intensity value of part of the vessel branches are also modified to simulate the situation of intensity inhomogeneity, which occurs in many medical images [13]. Figure 10(b)-(d) demonstrate the MIP images of the dataset corrupted by different Gaussian noises with variances of 0.001, 0.01 and 0.015, and parts of the vassel branches look like disconnected due to the effect of intensity inhomogeneity.

**Figure 11:**
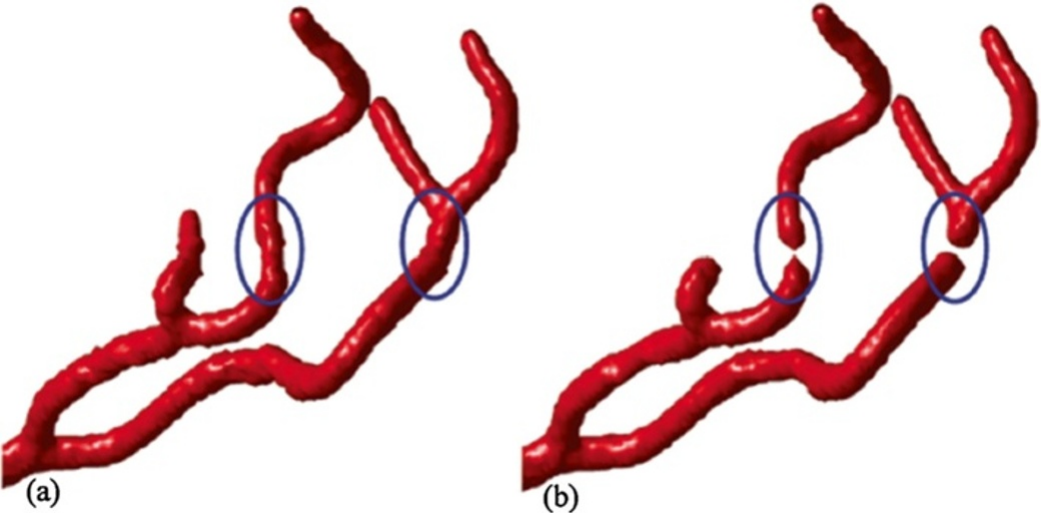
**The comparison of segmentation results obtained from noisy dataset between the proposed model (a) and the original model (b).**

In these experiments, for the purpose of comparison, we set the same seed contours for both the original and our localized models. Figure 11 shows the the segmentation results obtained from noisy dataset with variance of 0.001. Ellipse areas of panel (b) demonstrate that the original model is unable to extract the connected vessel branches due to the effect of intensity inhomogeneity, even though the global threshold indicating the lower bound of intensity value of target vessel is set very low. On the other hand, our proposed model can solve this problem by calculating the local threshold automatically according to the local image information, which can extract the connected vessel branches shown in ellipse areas of panel (a).

Besides the visual inspection, we calculate the segmentation errors using the Dice similarity coefficient [29] for quantitative validation:
10

where *A* and *B* are the target and obtained segmentation sets, and *n* is the voxel number. The smaller value of SE means the more accuracy of the segmentation result.

Table 1 illustrates the segmentation errors of Chan-Vese (CV) model [12], Region-Scalable Fitting Energy(RSFE) based method [14], Nonparametric Shape Prior Constrained Active Contour (NSPCAC) Model [24], Original Hybrid Model [4], and the proposed Localized Hybrid Model for different Gaussian noise variance levels applied on the test dataset. The other four methods that we compared with are typical: CV is a classical region based active contours model; RSFE is a well-known segmentation method that utilizes the local image information; NSPCAC is a recently developed method for vessel image segmentation; The Original Hybrid Model is the most relevant method to the proposed Localized Hybrid Model. As have been shown in the table, the SE values of our proposed method are smaller than that of CV model, RSFE based method, NSPCAC model, Original Hybrid Model for each different variance levels. That is, by keeping the advantages of hybrid level-set model as well as using locally specified dynamic thresholds, our method can achieve more accurate segmentation results than other methods.

**Table 1:** **Segmentation Errors of Chan-Vese (CV) model, Region-Scalable Fitting Energy (RSFE) based method, Nonparametric Shape Prior Constrained Active Contour (NSPCAC) model, Original Hybrid Model and Localized Hybrid Model for different Gaussian noise variance levels applied on the test dataset**

Methods	***σ***=0.001	***σ***=0.005	***σ***=0.01	***σ***=0.015
CV model	15.88 *%*	16.54 *%*	20.55 *%*	21.58 *%*
RSFE based method	12.26 *%*	15.20 *%*	17.33 *%*	19.58 *%*
NSPCAC model	9.80 *%*	12.19 *%*	14.15 *%*	16.66 *%*
Original Hybrid Model	8.70 *%*	9.03 *%*	9.78 *%*	10.65 *%*
Localized Hybrid Model	8.15 *%*	8.63 *%*	9.13 *%*	9.96 *%*

## Conclusions

Vessel image segmentation is an essential step for many clinical tasks such as diagnosis of vessel diseases, surgery planning and blood flow simulation. However, due to the high complexity of vessel branching and thinning geometry, blood vessel image segmentation still remains a challenging task. In this paper, a localized hybrid level-set model for vessel image segmentation is proposed. Our technique is inspired by Zhang et al.’s hybrid level-set model [4]. However, instead of using a preset global threshold to specify the term concerning regional information, the proposed method compute the local thresholds automatically to indicate the lower bound of target object, which is crucial for the segmentation of vessel images. Compared with the global threshold based method, the use of locally specified dynamic thresholds enables the extraction of the local image information. Our proposed method has been successfully applied to over ten 3D medical datasets for the segmentation of vasculatures. Some experimental results are presented to demonstrate the strengths of our localized hybrid level-set method. In addition, both the qualitative and the quantitative validations have been performed to indicate that our proposed model can achieve more accurate segmentation results than original hybrid model and CV model do.
